# A nationwide postal survey on the perception of Malaysian public healthcare providers on family medicine specialists’ (PERMFAMS) clinical performance, professional attitudes and research visibility

**DOI:** 10.1186/s40064-015-1004-9

**Published:** 2015-05-06

**Authors:** Boon-How Chew, Mazapuspavina Md Yasin, Ai-Theng Cheong, Mohd-Radzniwan A Rashid, Zuhra Hamzah, Mastura Ismail, Norsiah Ali, Baizury Bashah, Noridah Mohd-Salleh

**Affiliations:** Department of Family Medicine, Faculty of Medicine and Health Sciences, Universiti Putra Malaysia, 43400 Serdang, Selangor Malaysia; Primary Care Medicine Discipline, Level 7, Academic Block, Faculty of Medicine, UiTM Sg. Buloh Campus, Jalan Hospital, 47000 Sungai Buloh, Selangor Malaysia; Jabatan Perubatan Keluarga, Fakulti Perubatan, University Kebangsaan Malaysia, Jalan Yaacob Latif, Bandar Tun Razak, 56000 Kuala Lumpur, Malaysia; Klinik Kesihatan Seremban 2, Jalan S2 A2, 70300 Seremban 2, Negeri Sembilan Malaysia; Klinik Kesihatan Tampin, 73000 Tampin, Negeri Sembilan Malaysia; Klinik Kesihatan Putrajaya Precinct 9, 1, Jalan P9E, Presint 9, 62250 Putrajaya, Wilayah Persekutuan Putrajaya Malaysia; Bahagian Pembangunan Kesihatan Keluarga, Kementerian Kesihatan Malaysia, Aras 7 & 8, Blok E10, Kompleks E, Pusat Pentadbiran Kerajaan Persekutuan, 62590 Wilayah Persekutuan Putrajaya Malaysia

**Keywords:** Perception, Healthcare providers, Family physicians, Physician’s practice patterns, Clinical competence, Professional competence, Clinical ethics, Research activities, Malaysia

## Abstract

Perception of healthcare providers who worked with family medicine specialists (FMSs) could translate into the effectiveness of primary healthcare delivery in daily practices. This study examined perceptions of public healthcare providers/professionals (PHCPs) on FMSs at public health clinics throughout Malaysia. This was a cross-sectional study in 2012-2013 using postal method targeting PHCPs from three categories of health facilities, namely health clinics, health offices and hospitals. A structured questionnaire was developed to assess PHCP’s perception of FMS’s clinical competency, safety practice, ethical and professional values, and research involvement. It consists of 37 items with Likert scale of strongly disagree (a score of 1) to strongly agree (a score of 5). Interaction and independent effect of the independent variables were tested and adjusted means score were reported. The participants’ response rate was 58.0% (780/1345) with almost equal proportion from each of the three public healthcare facilities. There were more positive perceptions than negative among the PHCPs. FMSs were perceived to provide effective and safe treatment to their patients equally disregards of patient’s social background. However, there were some concerns of FMSs not doing home visits, not seeing walk-in patients, had long appointment time, not active in scientific research, writing and publication. There were significant differences in perception based on a respondent’s health care facility (*p* < 0.0001) and frequency of encounter (*p* < 0.0001). PHCPs had overall positive perceptions on FMSs across all the domains investigated. PHCPs from different health care facilities and frequency of encounter with FMSs had different perception. Practicing FMSs could improve on the critical service areas that were perceived to be important but lacking. FMSs might need further support in conducting research and writing for publication.

**Electronic supplementary material:**

The online version of this article (doi:10.1186/s40064-015-1004-9) contains supplementary material, which is available to authorized users.

## Background

Collaboration between family physicians and other specialists is of critical importance to the care of many patients (Cook et al. 2000; D’Amour et al. 2005; Frost et al. 2012). Thus, perception of healthcare providers, including the health clinic’s staff, who worked with family medicine specialists (FMSs) could affect the effectiveness of primary healthcare delivery on daily basis (Guldberg et al. 2009; Pinder et al. 2013; Stevenson et al. 2001). Working together among the health care professionals on behalf of patients requires teamwork that occurs across a complex set of inter-professional relationships and services (Martin et al. 2004). It requires skilful management of the relationships with appropriate authority in the collaboration and in need of vigilance for continuous process improvement (Martin et al. 2004). This care coordination and co-operation among clinicians were the priority areas for quality improvement in many clinical practices (Adams and Corrigan 2003; A Committee on Quality of Health Care in America 2001).

Primary medical care in this country is provided for by two main sectors, namely the private general practitioners (GPs) and the public health clinics (Jaafar et al. 2013). The formers are mostly in and around cities with service 100% paid out-of-pocket by the patients. Whereas, the public health clinics are distributed throughout the country, with the smaller clinics situated in the rural areas (Awin 2001; Yasin et al. 2012). The bigger public health clinics have resident doctors (non-specialists) and FMSs, and equipped with complete in-house medical facilities such as medical laboratory tests, plain x-rays and pharmacy. Established referral system is seamless for referral between the public health clinics and hospitals (Jaafar et al. 2013). In fact, FMSs are often involved in communication with the hospital specialists for patients who need secondary or tertiary care in hospitals. Health clinics are under the district health offices’ administration and coordination. FMSs have their official heads of department in the district health offices. Besides overseeing the running of all health clinics at the district level, district health officers and his/her public health professionals are managing community health issues. District health offices in every state come under the coordination and jurisdiction of a state health office, which is led by a state health director. Further details on the primary health care system and issues surrounding the FMSs’ practice had been published in an earlier report (Chew et al. 2014).

Despite its more than 30-years history of GPs’ and public primary medical care practices, almost 20-year of existent of the specialist training program for this specialty, there is a general lack of clear understanding of what the family medicine and its practice represent among the general public and the health care professionals. There were few studies reported on the perception of other healthcare professionals/providers at the health clinics, health offices and hospital specialists on FMSs. Realizing that something had to be done if the specialty was going to remain healthy, leaders of Family Medicine Specialists Association (FMSA), universities and Family Health Development Division (BPKK), Ministry of Health (MOH) together initiated this study to examine the perception of public healthcare providers/professionals (PHCPs) who have working relationships with the FMSs at the public health clinics. With the results of this study, we can gauge the performance and acceptance of FMSs as well as this specialty in the arena of primary health care. This information would also serve as a feedback to the relevant parties and stakeholders such as the FMSs themselves, FMSA, BPKK, MOH and the universities. Universities, colleges or academies that are training future FMSs can be better informed of the weaknesses or strengths of the current programs, thus improvement is possible for future FMSs to be better equipped to meet the market demands.

## Results

The centres response rate following the initial invitation was 40.0% (60/158). The participants’ response rate was 58.0% (780/1345). We receive almost equal proportion of completed questionnaires from each public healthcare facility (Table 1). Four states (Melaka, Sabah, Pahang and Johor) contributed almost half (47.6%) of the total responses, whereas Selangor, Putrajaya/Kuala Lumpur federal territories and Negeri Sembilan combined contributed about 10% of the total responses (Table 1). Table 2 shows that the mean age of participants at health clinics HCs was significantly younger compared to those at the other two facilities, and there were significantly more female participants at the non-hospitals compared to that at the hospitals. The frequency of more encounters with FMSs was seen at the HCs, followed by health offices HOs staff. The mean length in medical service was all significantly different among the three healthcare facilities where the HOs’ PHCPs had the longest and the hospital had the shortest. However, there was no different in term of length of service at the current facility among the three healthcare facilities.

**Table 1:** **Number (%) of participants according to the public healthcare facilities and the states**

**States**	**Public healthcare facility, n (%)**			**Total, n (%)**
	**Health clinics**	**Health offices**	**Hospitals**	
Sabah	59 (22.4)	14 (5.2)	24 (9.6)	97 (12.4)
Sarawak	20 (7.6)	36 (13.5)	0	56 (7.2)
Perlis	0	16 (6.0)	26 (10.4)	42 (5.4)
Kedah	0	15 (5.6)	17 (6.8)	32 (4.1)
Penang	14 (5.3)	14 (5.2)	5 (2.0)	33 (4.2)
Perak	17 (6.5)	14 (5.2)	20 (8.0)	51 (6.5)
Kelantan	20 (7.6)	15 (5.6)	12 (4.8)	47 (6.0)
Terengganu	40 (15.2)	28 (10.5)	0	68 (8.7)
Selangor	20 (7.6)	0	0	20 (2.6)
Negeri Sembilan	0	30 (11.2)	0	30 (3.8)
Melaka	37 (14.1)	14 (5.2)	53 (21.2)	104 (13.3)
Pahang	54 (19.2)	15 (6.0)	21 (8.4)	90 (11.5)
Johor	0	25 (9.4)	56 (22.4)	81 (10.4)
WP Putrajaya/Kuala Lumpur	0	13 (4.9)	16 (6.4)	29 (3.7)
**Total**	**263 (33.7)**	**267 (34.2)**	**250 (32.1)**	**780 (100.0)**

**Table 2:** **Socio-demography**

**Socio-demography**	**Total**	**Public healthcare facility**			***X*** ^**2**^ */F ^**†**^	**P value**
		**Health clinics**	**Health office**	**Hospitals**		
Age, mean (SD)	38.2 (8.83)	36.3 (9.25)^‡^	38.9 (9.87)^‡^	39.4 (6.78)^‡^	9.48	<0.0001
Gender						
Male	282 (100.0)	84 (29.8)	62 (22.0)	136 (48.2)	51.94	<0.0001
Female	485 (100.0)	191 (39.4)	181 (37.3)	113 (23.3)		
**Total**	**767 (100.0)**	**275 (35.9)**	**243 (31.7)**	**249 (32.5)**		
Profession						
Senior Consultants	26 (100.0)	0	0	26 (100.0)	792.37	<0.0001
Consultants	55 (100.0)	0	1 (1.8)	54 (98.2)		
Clinical Specialists	138 (100.0)	1 (0.7)	0	137 (99.3)		
Public Health Physicians	11 (100.0)	1 (9.1)	10 (90.9)	0		
Medical Officers	131 (100.0)	46 (35.1)	65 (49.6)	20 (15.3)		
Assistant Medical Officer	68 (100.0)	41 (60.3)	27 (39.7)	0		
Pharmacists	36 (100.0)	33 (91.7)	3 (8.3)	0		
Matrons & Sisters	57 (100.0)	17 (29.8)	40 (70.2)	0		
Staff Nurses	82 (100.0)	50 (61.0)	31 (37.8)	1 (1.2)		
Community Nurses	49 (100.0)	28 (57.1)	20 (40.8)	1 (2.0)		
Nutritionists & Dieticians	15 (100.0)	5 (33.3)	10 (66.7)	0		
Physiotherapists & Occupational therapists	6 (100.0)	6 (100.0)	0	0		
Medical Laboratory Technicians	20 (100.0)	20 (100.0)	0	0		
Radiographers	8 (100.0)	8 (100.0)	0	0		
Others	63 (100.0)	22 (34.9)	39 (61.9)	2 (3.2)		
**Total**	**765 (100.0)**	**278 (36.3)**	**246 (32.2)**	**241 (31.5)**		
Average number of encounter with FMS						
Almost every working day	96 (100.0)	53 (55.2)	41 (42.7)	2 (2.1)	209.27	<0.0001
1-3 times per week	150 (100.0)	84 (56.0)	54 (36.0)	12 (8.0)		
1-3 times per month	182 (100.0)	65 (35.7)	74 (40.7)	43 (23.6)		
1-6 times per year	203 (100.0)	30 (14.8)	39 (19.2)	134 (66.0)		
< 6 times per year	46 (100.0)	17 (37.0)	15 (32.6)	14 (30.4)		
Never	4 (100.0)	2 (50.0)	2 (50.0)	0		
**Total**	**681 (100.0)**	**251 (36.9)**	**225 (33.0)**	**205 (30.1)**		
Length in medical service (year), mean (SD)	11.4 (9.01)	11.3 (8.68)^§^	14.1 (10.05)^§^	8.8 (7.40)^§^	22.59	<0.0001
Length of service in current health facility (year), mean (SD)	5.2 (5.33)	4.9 (5.06)	5.3 (5.69)	5.5 (5.24)	1.00	0.39

*Chi-square test, ^†^ANOVA.

^‡^Post-hoc Bonferroni showed that mean age at health clinics was significantly younger compared to the others, no different was seen between health office and hospital.

^§^Post-hoc Bonferroni showed that mean length in medical service was all significantly different among the three healthcare facilities.

**Figure 1:**
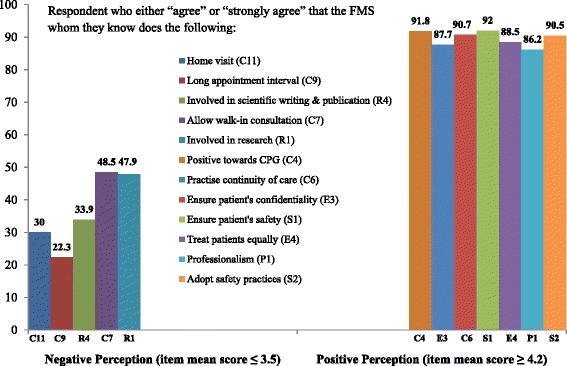
Proportion of responses indicating some of the most negative and positive perception by the PHCP towards family medicine specialists according to the means of responses. PHCP = public healthcare providers, CPG = clinical practice guideline.

Generally, there was more positive perception than negative among the PHCPs towards the FMSs [see Additional file 1]. More than 80% of respondent surveyed “agreed” or “strongly agreed” that FMSs did provide appropriate medical management that improved patients overall health. FMSs practised continuity of care (often see back/follow-up his/her patients), were positive towards clinical practice guidelines and delivered evidence-based care. Almost 90% “agreed” or “strongly agreed” that FMSs safeguarded patient’s confidentiality and treated them equally irrespective of social background; FMSs ensured patient safety in treatment and adopted safety practices (e.g. universal precaution) at the clinics. However, there were some concern of FMSs seeing walk-in patients and long appointment time for those in need of FMSs care (Figure 1). About 20% of the respondent perceived FMSs as making inappropriate referral to the hospitals, pre-referral preparation and writing inappropriate referral letters.

**Figure 2:**
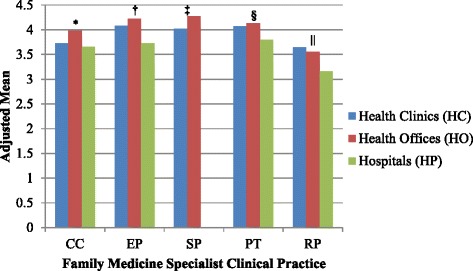
Adjusted means of cumulative scores in clinical competency, ethical practice, safe practice, professionalism & team work and research & publication according to the different health care facilities after controlling for encounter. CC = clinical competency, EP = ethical practice, SP = safety issue in clinical practice, PT = professional attitude and team-working, RP = research collaboration and publication, Health Facilities = health clinics, health offices and hospitals. Post-hoc Bonferroni multiple comparisons: *HO>HC>HP. †HO = HC>HP, HO>HP. ‡HO>HC. §HO = HC>HP, HO>HP. ||HO = HC>HP, HO>HP.

**Figure 3:**
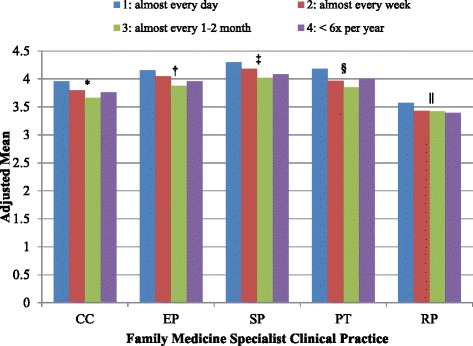
Adjusted total means of cumulative scores in clinical competency, ethical practice, safe practice, professionalism & team work and research & publication according to the different encounter after controlling for health care facilities. CC = clinical competency, EP = ethical practice, SP = safety issue in clinical practice, PT = professional attitude and team-working, RP = research collaboration and publication. Encounter comprises four categories: 1: is almost every day; 2: is almost every week; 3: is almost every 1-2 month; 4: is < 6x per year. Post-hoc Bonferroni multiple comparisons: *1>2>3, 1>4, 2 = 4, 3 = 4. †1>2>3, 1>4, 2 = 4, 3 = 4. ‡1 = 2 = 3 = 4. 1>3, 1 = 4, 2 = 4. §1>2>3<4, 1>3, 1 = 4, 2 = 4. ||1>2>3 = 4, 1>3, 1>4, 2 = 4.

Table 3 shows there was a statistically significant difference between adjusted cumulative mean score of each perception on the FMSs’ clinical practice based on respondents’ health care facility (*p* < 0.0001). PHCPs at hospitals HPs generally had more negative perceptions on FMSs compared to those at HCs and HOs (Figure 2). PHCPs at HOs tend to have similar (EP, PT and RP) or better (CC and SP) perception on FMSs’ clinical practices compared to those at the HCs. There was also a statistically significant difference between adjusted cumulative mean score of most perceptions on the FMSs clinical practices based on respondents’ number of past encounters with FMSs (*p* < 0.0001) except in RP (Table 3). PHCPs who had more frequent encounter (almost every day) were generally had more positive perception on FMSs compared to other frequencies of encounter (Figure 3). The effect sizes of the health care facility were larger than those of the encounter across all the dependent variables (CC, EP, SP, PT and RP).

**Table 3:** **Analysis of covariance (ANCOVA) presenting the independent and main effect of health care facilities and encounter on each of the total means perception of family medicine specialist’s clinical practice**

**Source**	**Type III sum of squares**	**Mean square**	**F**	**Sig.**	**Partial eta squared**	**Adjusted R squared**
**Dependent Variable: Total Mean CC, n = 681**						
Health Facilities	11.381	5.691	25.713	<0.0001	0.071	0.156
Encounter	7.604	2.535	11.453	<0.0001	0.048	
**Dependent Variable: Total Mean EP, n = 677**						
Health Facilities	20.386	10.193	29.397	<0.0001	0.081	0.170
Encounter	6.063	2.021	5.828	<0.0001	0.025	
**Dependent Variable: Total Mean SP, n = 473**						
Health Facilities	7.142	7.142	22.107	< 0.0001	0.045	0.060
Encounter	5.103	1.701	5.266	< 0.0001	0.033	
**Dependent Variable: Total Mean PT, n = 681**						
Health Facilities	9.774	4.887	12.843	< 0.0001	0.037	0.124
Encounter	9.385	3.128	8.221	< 0.0001	0.035	
**Dependent Variable: Total Mean RP, n = 670**						
Health Facilities	20.165	10.082	22.823	< 0.0001	0.064	0.113
Encounter	2.839	0.946	2.143	0.094	0.010	

CC = clinical competency, EP = ethical practice, SP = safety issue in clinical practice, PT = professional attitude and team-working, RP = research collaboration and publication.

Health Facilities = health clinics, health offices and hospitals.

Encounter comprises four categories: 1: is almost every day; 2: is almost every week; 3: is almost every 1–2 month; 4: is < 6x per year.

## Discussion

This study had been able, for the first time in this country, to collect perception on the public FMSs in many significant domains of clinical practices from PHCPs of almost all categories, at the three main health care facilities, in a nationwide cross-sectional questionnaire-based study. We found that there was an overall positive perception on FMS professionalism, clinical competency and ethical practice. FMS were perceived to provide effective and safe treatment to their patients; equal care was provided disregard of patient’s social background, and FMSs were perceived to safeguard patient’s confidentiality. While some of these clinical attributes were perceived to be outstanding in the FMSs, the results also showed areas of concerns such as in doing home visit, patient referral, walk-in consultation and appointment interval.

### Clinical performance and professional attitudes

The practice of home visit and its relevance might need to be locally determined. In the public primary medical care services where FMSs was relatively scarce, restricting FMSs home visit to the really necessary patients while delegating others to the paramedics could have been the contemporary practice. Although such a task delegation could save the FMSs working hours to see extra patients per month (van den Berg et al. 2012), this task delegation and distribution among the clinic staff should be clearly communicated to the concerned staff so to avoid misunderstanding and mal-perception. Probably similar vigilance was needed when referring patients for hospital care, which the decision might arise amidst a busy clinic. Referral process was often a complex decision arrived at owing to many causes and it was exposed to hospital specialists’ asymmetric judgement (Thorsen et al. 2012). Differences in individual FMS expertise, access to resources, local styles of practices, FMS’s relationship with hospital specialists/consultants could be important factors affecting referral decisions and the perceived quality of referral letters (Chan 1999; Langley et al. 1997). Having a referral guideline, incorporating structured referral sheets, and requiring a second ‘in-house’ opinion prior to referral might increase thoughtfulness in referral practice that would help in improving outpatient referrals from primary care to secondary care (Faulkner et al. 2003; Frost et al. 2012; Vachon et al. 2013). Having hospital consultants to provide their service at the health clinics, to reply referral letters from the primary care physicians/FMSs and involved in educational activities at the health clinic could help to improve the quality of referrals (Akbari et al. 2008).

FMSs’ appointment system was perceived to have too long waiting time (defined as more than one month) for new patients. We could not determine its appropriateness based on the present study but the clinic staff’s perception could reflect the general reaction of the patients upon given the appointment. Timely access has been rated by patients as one of the most important elements of primary care especially when FMS’s care has been deemed necessary by other primary health care professionals (Wong et al. 2008). The FMS’s appointment system should be able to meet patients’ requests on the same day the request is being made (The College of Family Physicians of Canada 2012). This immediate medical attention would either allow the FMS to decide on the length of appointment to be given or even deliver the needed care there-and-then which would be appreciated by patient with urgent conditions. Same-day scheduling could build trust, reinforce the patient-physician relationship and decrease the number of no-shows (Mitchell 2008). Same-day scheduling, also known as advanced access and open access, is an appointment system that do “today’s work today” by confirming with patients their appointments on the day they request it (The College of Family Physicians of Canada 2012).

### Research visibility

This study suggested that while many FMSs were conducting medical audits and providing feedback to their health clinics staff, less than half of the respondent perceived FMSs being involved in medical research and writing for publication. Every FMS who had graduated from any residency programs in Malaysia had in fact successfully conducted and completed a medical research. Abstracts of their studies might have been presented in scientific conferences. However, owing to the many duties and responsibilities of a public FMS, many might not be able to publish previous works or initiate new research projects (Weber-Main et al. 2013). It was reported that even in the United States only about one-thirds of all the abstracts presented in family medicine and primary care conferences were actually published (Post et al. 2013). Thus, FMSs would need to enhance personal motivation, professional skills in research collaboration with other FMSs or academic FMSs in research project, scientific writing and publication. They may require practical support such as protected time, incentives and rewards in conducting and publishing researches in order to increase their research expertise and visibility (Weber-Main et al. 2013).

### Challenges in the family practice

Negative perception of family physicians and their practice by other health professionals especially the hospital specialists were not uncommon elsewhere (Kamien et al. 1999; Manca et al. 2008; Miedema et al. 2012). Overseas family physicians were reported to feel as if there was a hierarchical relationship with the hospital specialists, and they were at the lower part of the top-down relationship (Thorsen et al. 2012). Some features of general practice such as seeing undifferentiated diseases, being the first contact for many re-emerging diseases, providing comprehensive care to a whole family, an increasing pluralistic and health literacy challenging society produce more frequent medical uncertainties and confrontations with ethical issues than are encountered in other disciplines (Ariff and Beng 2006; Kaur 1994; Smith 1987). These characteristics of FMS’s practice could easily be misunderstood as lacking in clinical competency, unnecessary referral, unprofessional or unethical practices. Malaysian public health clinics were reported to see a total of about 28 millions out-patients per year in 2011 compared to about two millions in-patients in the public hospitals (Ministry of Health Malaysia 2011). This high number of patient visit and wide variety of medical conditions might impose a great challenge to the practising FMSs to maintain a high standard of care for all the patients managed by primary care professionals under his/her supervision at the health clinics (Chew et al. 2012; Kamarudin et al. 2012; Omar et al. 2011). Time pressure in primary care medical consultation had been related to non-adherence to clinical practice guideline, insufficient history taking and advice on lifestyle changes to patients with respiratory tract infections (Tsiga et al. 2013). In chronic diseases (chronic angina, bronchial asthma and type 2 diabetes) management, Campbell et al showed that the most powerful predictor of quality care for these diseases in the English general practices was the length of the consultation (Campbell et al. 2001).

Despite high work-load, family medicine was being affected by a combination of other factors: fundamental inadequacies in the Malaysian health care system that include inappropriate staff organization, inadequate staffing and financing arrangements, unprecedented knowledge and technological advances, and mismatches between what was needed and wanted by hospital PHCPs and what FMSs were positioned to provide (Chan 1999; Noor Ghani and Yadav 2008; Yasin et al. 2012). Health system was perceived by the GPs in the Republic of Ireland to be both barriers and facilitators for primary care management of many diseases especially diabetes mellitus, and health system’s effect rippled at an organisational, professional and patient level (Mc Hugh et al. 2013). Nevertheless, this study had provided an intriguing perspective of those PHCPs who have more encounters with FMSs, were actually holding more positive perception towards FMSs and their clinical practices. It was highly possible that more frequent and closer working relationship with a FMS facilitate a truer understanding about the FMSs and their clinical practices among the PHCPs across the different health care facilities (Sargeant et al. 2008).

### Implications and recommendations

New approaches to lifelong learning should be effective to keep FMSs abreast of updated medical knowledge in order to manage their patients with high standard of care, make appropriate referrals; office procedures skills training that enable FMS to perform relevant procedures and more adept in using office investigative tools/technologies (Manca et al. 2008; Martin et al. 2004). More importantly, family medicine training programs in the local universities and other post-graduate programs for family medicine fraternity to make relevant changes to meet the expectations of their colleague at primary care and higher care levels (Manca et al. 2008). BPKK of the MOH would want to provide conducive primary care system that is well supported by adequate resources and investment in community services, has serendipitous access to secondary services and vocational incentives for its healthcare providers/FMSs in order to prevent apathy (Mc Hugh et al. 2013). Equally important is a better understanding of family medicine specialty by PHCPs for truer perception, such as in managing undifferentiated symptoms and uncertain medical conditions require unique skills in family medicine that should not be alluded to as incompetency. Hence, more efforts and opportunity should be in place to encourage PHCPs to have more cognizance of public family medicine practice, which would enhance mutual understanding that could benefit the current healthcare delivery nationwide.

### Strength and limitation

Some of the strengths of this study include the nationwide coverage of PHCPs from the three different public healthcare facilities. We caution and suggest state-level or district-level studies for a more precise perception due to increasing disproportionate representation of the respondents at these levels. We encouraged voluntary participation at the health care facilities levels in order to improve feasibility, but it could have been a universal sampling in many small health care facilities such as the district hospitals. Voluntary participation might invite exceedingly favourable or unfavourable responses. Although both types of responses were expected in this survey but we were uncertain of the true proportion of them in this study. Nevertheless, the result of less favourable perception from the hospitals’ PHCPs was in line with many FMSs’ experiences. Therefore, we believe this survey had served well the purpose of confirming the perception of the PHCPs on FMSs. Structured questionnaire used in this study could have missed other factors deemed important for the different perception variables. Therefore, further qualitative study on key informants is needed to discover other important aspects of the FMSs’ clinical practice. For this purpose, this study had indicated that hospital-based PHCPs would be a suitable choice of informants to explore further on FMSs’ areas for improvement; while the health office-based PHCPs would provide the FMSs’ desirable attributes to be reinforced. In addition, it is desirable to have patient’s perception on the current health care system where FMSs are working to complement the findings of this study.

## Conclusions

Perception of PHCPs who worked closely with FMS was an essential piece of feedback information to primary health care in this country. This study had provided an opportunity for other primary healthcare providers, hospital specialists and administrative staff at the health offices to feedback on the FMSs’ roles and performance. It had also provided family medicine specialty and specialists an invaluable source of data for self-reflection. We reported that PHCPs had overall positive perception on FMS across all the domains investigated. PHCPs from non-hospital health care facilities and who had higher frequency of encounter with FMSs had more positive perceptions. Discrepancy between perceptions and the relevance of certain clinical practices such as home visit may need to be communicated locally. Having shorter appointment interval and allowing walk-in consultation would be the areas of improvement in FMSs’ family practice. FMSs might need support to increase their research visibility by involving more in research activity at the local clinic/community levels and contribute to scientific writing and publication.

## Methods

This was a cross-sectional study using postal method throughout Malaysia targeting PHCPs from three categories of health facilities, namely health clinics (HC), health offices (HO) and hospitals (HP).

### Ethics statement

This study was approved by the Medical Research Ethics Committee (MREC) on Dec 2008. Protocol Number from the National Medical Research Register: NMRR ID: 08-12-1167. Participants provided verbal consents before completing the questionnaire.

### The setting

We invited every state’s general hospitals and one district hospital from each state (list of states is shown in Table 1). The district hospitals chosen were those with which FMSs had much working relationships with and this decision was made after discussion with the state FMSs’ representatives/heads. Using Excel random number generator, we randomly selected five health clinics from every state based on the 2010 directory of health clinics with resident FMSs. In the states with less than five clinics, all these clinics were chosen. We invited every state’s health offices. District health offices that have their health clinic/s selected within their districts were also invited.

### The subjects

PHCPs were invited to participate voluntarily. Having previous personal encounters with FMSs was emphasized in the information sheet. Encounter was defined as contacts through referral letters, direct consultation in-person or via telephone, emails, in official or scientific meetings. At the hospitals, only doctors were invited with priority for clinical specialists and consultants, house officers were excluded. All categories of PHCPs at the health clinics and health offices were invited except house officers, health attendant and those who had psychiatric disorders that impair judgement and memory.

### The sampling

**Figure 4:**
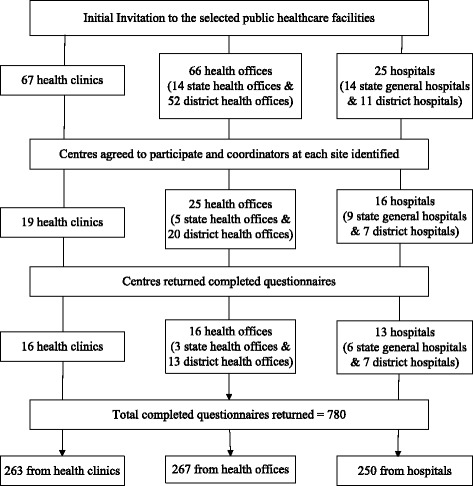
The study progression chart.

Samplings comprised initial invitation to all the selected public healthcare facilities and followed by directed posting of the questionnaires (Figure 4). At the participating centres, convenient sampling of participants was used. The initial invitation document-package was sent to the heads of the healthcare facilities consisting of an endorsement letter from the director of the BPKK, the letter of MREC approval, a letter from the principle investigator, the information sheet for site-coordinators with a study flow-chart and a reply form. Two main purposes of this initial invitation were to encourage participation from the selected centres; and second was to elect a dedicated site-coordinator at the participating centres to distribute and recollect questionnaires. We requested personal particulars and contact details of the site-coordinator and set a final date in the reply form. The selected centres were to fax back the reply forms to the principle investigator to indicate their willingness to participate. FMSs at the health clinics were avoided throughout the sampling process.

Following the return of their reply forms, the decided numbers of questionnaires were posted by courier service directed to the site-coordinators at the participating centres. Site-coordinators distributed the questionnaires according to the set criteria. After collecting back the completed questionnaires and sealing them in the provided courier envelopes, site-coordinators informed investigator by faxing a notice form for recollection of the questionnaires by courier service that reverse-charged the investigator.

### The instrument

We developed a structured questionnaire consists of 37 items with Likert scale of strongly disagree (a score of 1) to strongly agree (a score of 5) and two open-ended items asking respondent’s general impression and expectation of FMSs. The items C1 to C17 assess clinical competency and adherence to evidence-based medicine (CC). The items E1 to E4 gauge ethical practices (EP), items S1 to S4 evaluate safety issue in clinical practice (SP) and items P1 to P8 assess professional attitude and team working (PT), items R1 to R4 assess FMS involvement in research collaboration and scientific writing and publication (RP). These items were based on a review of literature (Adams and Corrigan 2003; A Committee on Quality of Health Care in America 2001; Haddad et al. 2000; Schwarz et al. 1999; Stevenson et al. 2001; Wong et al. 2008) and where it was lacking, the expert consensus among the team members of investigators were consulted, to capture the varied concepts of clinical practices. The questionnaire for the health clinics’ PHCPs had all these items [see Additional file 1] but these items were reduced to that were thought appropriate for PHCPs at hospitals and health offices. Hospitals’ PHCP had C1-3, C5, C12-13, C14 and C16-17; E1-2; P1-P4, P7; R1 and R4; and no SP items. Health offices’ PHCP had C1, C3-4, C12-13, C15; E1-4; S1-2; P1-8; R1 and R4. The questionnaire was developed in English; it was then back-to-back translated into Malay. Each of these questionnaires was tested for face and content validity with 10 PHCPs from each healthcare facility and questionnaire was further improved based on their feedback. The English version was used for hospital specialists whereas the Malay version was used for PHCPs at health clinics and health offices. We included a copy of the questionnaire in the other language to all the participating centres in order to serve as a cross reference for healthcare professionals who may be more proficient in the other language. The internal consistency (Cronbach’s Alpha) for the items according to their domains from each of the healthcare facility ranges from 0.77-0.89 (CC), 0.86-0.89 (EP), 0.86-0.89 (SP), 0.90-0.95 (PT) and 0.75-0.94 (RP) [see Additional file 2].

Every questionnaire was clearly printed in an A4 size booklet that flips open towards the left. It begins with an information sheet to the participant, demographic data, the items with Likert scale and ends with two open-ended items. They were coded according to the state and category of health facility. The state code used the Road Transport Department coding system for vehicles and we coded C for clinics, A for health offices and H for hospitals. Confidentiality of the participant was maintained whereby no personal particulars such as name and staff identification number were asked. Privacy of the responses to the questions was further guarded by asking the respondent to staple at least on the six sites, as indicated, around the questionnaire booklet.

### Sample size

Sample size calculation was done using Epi Info 3.5.1. The estimated number of PHCPs from 25 public hospitals (14 general hospitals and 11 district hospitals), 67 public health clinics and 66 health offices was 5,000 people. We expected the poor perception of FMSs at 10% with worst scenario of 5%; the sample size was 228 with 99% confidence interval. After taking into consideration of response rate of 50% and 30% of incomplete questionnaires, the needed sample size was 1140.

With this estimated sample size, we decided that each state general hospital was to receive 50 questionnaires, each district hospital 20, each health clinic 10 and each health office 10. This would provide a total of 2250 and of about equal proportion from each category of centres. However, owing to lesser than expected responses to participate from health clinics and health offices (Figure 4), we increased the number of questionnaires to health clinic to 20 and health office to 15. With these numbers of participants, it is estimated that more than half of the defined PHCPs at each healthcare facilities could be recruited. In a few district hospitals with lesser than 20 specialists, we included medical officers with preferences to those who were more senior and/or had been working there for longer number of years.

### Statistical analyses

Descriptive analyses were carried out using PASW 21.0 (SPSS, Chicago, IL). We collapsed the encounter variable from the initial six categories into four so to improve distribution of cases for statistical purposes: the first is almost every day; second is almost every week; third is almost every 1-2 month and the fourth is < 6x per year. In this report, we looked into the differences of perception between the PHCPs of the three main different health care facilities (HC, HO and HP) and not the 5-categories work-place variable (health clinic, state health office, district health office, state general hospital and district hospital), not also the professions, which would be reported separately. We used the duration of service at current profession instead of duration of service at current work place or age as they correlate significantly, r = 0.49 and r = 0.81 respectively, because the duration of service at current profession has more meaningful interpretation.

Comparisons of mean levels were performed using the Student’s t test and ANOVA test to determine the association between the socio-demographic data and the FMSs’ clinical practice. After entering all the independent variables in this study in the univariable analyses, we found that only health care facility and encounter variables had significant effects on the cumulative means CC, EP, SP, PT and RP. Two-way analysis of variance was used to test the interaction effect of these two independent variables, on each of the cumulative means of the perception variables (CC, EP, SP, PT and RP), and there were no interaction effects between these two variables. Analysis of covariance (ANCOVA) was used to present the independent and main effects of the different levels of the health care facility and encounter to produce the adjusted means. Post-hoc Bonferroni multiple comparisons were done for the different health care facilities and different categories of encounters. A *P* value < 0.05 was considered to be significant at two tails. Statistical assumptions for the both the two-way analysis of variance and ANCOVA in terms of the normality of the dependent variable and residuals, and equality of variances were confirmed. There was no significant outlier.

## Additional files

Additional file 1:
**Number (%) of each responses and mean (SD) of the score for each of the items.**
Additional file 2:
**Internal Consistency (Cronbach’s Alpha) for the Items according to the Domains and Public Healthcare Facilities.**

## Abbreviations

ANCOVA: Analysis of covariance
BPKK: Family Health Development Division
CC: Clinical competency
EP: Ethical practices
FMSs: Family medicine specialists
FMSA: Family Medicine Specialists Association
GPs: General practitioners
HC: Health clinics
HO: Health offices
HP: Hospitals
MOH: Ministry of Health
MREC: Medical Research Ethics Committee
PHCPs: Public healthcare providers/professionals
PT: Professional attitude and team working
RP: Research collaboration and scientific writing and publication
SP: Safety issue in clinical practice
