# Supplementary material for: A nationwide postal survey on the perception of Malaysian public healthcare providers on family medicine specialists’ (PERMFAMS) clinical performance, professional attitudes and research visibility
Source: Springerplus. 2015 May 6;4:213. doi: 10.1186/s40064-015-1004-9 (PMC4431984; doi:10.1186/s40064-015-1004-9)
Supplement: Additional file 1: — Number (%) of each responses and mean (SD) of the score for each of the items. [file 40064_2015_1004_MOESM1_ESM.docx]

**Additional file 1: Number (%) of each responses and mean (SD) of the score for each of the items**

| **Items** | | Total, n | n (%) | | | | | Mean (SD) | | | |
| --- | --- | --- | --- | --- | --- | --- | --- | --- | --- | --- | --- |
|  |  |  | Strongly Disagree | Disagree | Neither Agree Nor Disagree | Agree | Strongly Agree | Total | HC | HO | HP |
| C1 | Improve the overall health of patients under his/her care. | 776 | 1 (0.1) | 9 (1.2) | 86 (11.1) | 459 (59.1) | 221 (28.5) | 4.2 (0.66) | ***4.3 (0.66)*** | ***4.4 (0.56)*** | ***3.8 (0.62)*** |
| C2 | Manage patient appropriately. | 525 | 1 (0.2) | 12 (2.3) | 88 (16.8) | 327 (62.3) | 97 (18.5) | 4.0 (0.68) | ***4.1 (0.69)*** | NA | ***3.8 (0.68)*** |
| C3 | Practise evidence-based care for his/her patients | 771 | 0 | 9 (1.2) | 139 (18.0) | 426 (55.3) | 197 (25.6) | 4.1 (0.69) | ***4.2 (0.62)*** | ***4.3 (0.58)*** | ***3.6 (0.66)*** |
| C4 | Have a positive mind-set towards clinical practice guidelines. | 526 | 1 (0.2) | 2 (0.4) | 40 (7.6) | 282 (53.6) | 201 (38.2) | 4.3 (0.64) | ***4.2 (0.68)*** | ***4.4 (0.58)*** | NA |
| C5 | Empower patients to involve in their own care | 520 | 1 (0.2) | 12 (2.3) | 169 (32.5) | 266 (51.2) | 72 (13.8) | 3.8 (0.72) | ***4.0 (0.69)*** | NA | ***3.8 (0.72)*** |
| C6 | Practice continuity of care (often see back/follow-up his/her patients) | 277 | 1 (0.4) | 1 (0.4) | 32 (11.6) | 135 (48.7) | 108 (39.0) | 4.3 (0.70) | 4.3 (0.70) | NA | NA |
| C7 | Allow walk-in consultation from patient | 278 | 8 (2.9) | 32 (11.5) | 103 (37.1) | 96 (34.5) | 39 (14.0) | 3.5 (0.97) | 3.5 (0.97) | NA | NA |
| C8 | Allow ad-hoc consultation from MOs/allied health personals | 273 | 0 | 5 (1.8) | 71 (26.0) | 145 (53.1) | 52 (19.0) | 3. 9 (0.72) | 3.9 (0.72) | NA | NA |
| C9 | Have long waiting time for new referral (more than one month) | 278 | 13 (4.7) | 91 (32.7) | 112 (40.3) | 56 (20.1) | 6 (2.2) | 3.2 (0.88)* | 3.2 (0.88) * | NA | NA |
| C10 | Does perform relevant procedures i.e. IUCD, Paps smear, ultrasound etc. | 275 | 6 (2.2) | 19 (6.9) | 92 (33.5) | 110 (40.0) | 48 (17.5) | 3.6 (0.92) | 3.6 (0.92) | NA | NA |
| C11 | Home visit to needy patient | 276 | 20 (7.2) | 62 (22.5) | 111 (40.2) | 63 (22.8) | 20 (7.2) | 3.0 (1.02) | 3.0 (1.02) | NA | NA |
| C12 | Often receive compliment from patients | 766 | 8 (1.0) | 42 (5.5) | 394 (51.4) | 249 (32.5) | 73 (9.5) | 3.4 (0.78) | ***3.6 (0.80)*** | ***3.6 (0.78)*** | ***3.1 (0.63)*** |
| C13 | Often receive complaints from patients | 767 | 92 (12.0) | 215 (28.0) | 346 (45.1) | 94 (12.3) | 20 (2.6) | 3.4 (0.93)* | ***3.3 (1.00)*** * | ***3.6 (1.02)*** * | ***3.2 (0.67)*** * |
| C14 | Make appropriate pre-referrals preparation of patient for what to expect at the hospital | 526 | 6 (1.1) | 34 (6.5) | 146 (27.8) | 265 (50.4) | 75 (14.3) | 3.7 (0.83) | ***3.9 (0.74)*** | NA | ***3.4 (0.83)*** |
| C15 | Manage their time efficiently | 527 | 8 (1.5) | 23 (4.4) | 126 (23.9) | 264 (50.1) | 106 (20.1) | 3.8 (0.85) | 3.8 (0.94) | 3.9 (0.74) | NA |
| C16 | Refer appropriate cases for further management (timely, work-up etc.) | 247 | 0 | 12 (4.9) | 59 (23.9) | 152 (61.5) | 24 (9.7) | 3.8 (0.69) | NA | NA | 3.8 (0.69) |
| C17 | Write appropriate referral letter (adequate information, attachment etc.) | 247 | 1 (0.4) | 18 (7.3) | 66 (26.7) | 141 (57.1) | 21 (8.5) | 3.7 (0.75) | NA | NA | 3.7 (0.69) |
|  | **Clinical Competency** |  |  |  |  |  |  | **3.8 (0.53)** | **3.8 (0.50)** | **4.0 (0.49)** | **3.5 (0.49)** |
| E1 | Uphold patient welfare | 773 | 2 (0.3) | 10 (1.3) | 171 (22.1) | 413 (53.4) | 177 (22.9) | 4.0 (0.73) | ***4.1 (0.72)*** | ***4.2 (0.65)*** | ***3.6 (0.66)*** |
| E2 | Respect for patient’s autonomy/decision | 772 | 2 (0.3) | 13 (1.7) | 177 (22.9) | 429 (55.6) | 151 (19.6) | 3.9 (0.72) | ***4.0 (0.76)*** | ***4.2 (0.67)*** | ***3.7 (0.61)*** |
| E3 | Ensure patient’s confidentiality | 527 | 0 | 1 (0.2) | 48 (9.1) | 254 (48.2) | 224 (42.5) | 4.3 (0.65) | 4.3 (0.67) | 4.4 (0.61) | NA |
| E4 | Treat all patients equally irrespective of their social status | 529 | 3 (0.6) | 8 (1.5) | 50 (9.5) | 264 (49.9) | 204 (38.6) | 4.2 (0.73) | 4.2 (0.78) | 4.3 (0.68) | NA |
|  | **Ethical Practice** |  |  |  |  |  |  | **4.0 (0.65)** | **4.1 (0.64)** | **4.3 (0.56)** | **3.6 (0.59)** |
| S1 | Ensure patient safety in treatment | 525 | 1 (0.2) | 1 (0.2) | 40 (7.6) | 302 (57.5) | 181 (34.5) | 4.3 (0.62) | ***4.2 (0.65)*** | ***4.3 (0.57)*** | NA |
| S2 | Adopt safety practices (e.g. universal precaution) at the clinic | 527 | 1 (0.2) | 3 (0.6) | 46 (8.7) | 310 (58.8) | 167 (31.7) | 4.2 (0.63) | ***4.2 (0.68)*** | ***4.3 (0.57)*** | NA |
| S3 | Provide clinical input to rectify events as in incident reports | 278 | 2 (0.7) | 6 (2.2) | 54 (19.4) | 145 (52.2) | 71 (25.5) | 4.0 (0.78) | 4.0 (0.78) | NA | NA |
| S4 | Implementing measures to improve patient’s safety (checklists etc.) | 276 | 1 (0.4) | 8 (2.9) | 62 (22.5) | 151 (54.7) | 54 (19.6) | 3.9 (0.75) | 3.9 (0.75) | NA | NA |
|  | **Safe Practice** |  |  |  |  |  |  | **4.2 (0.59)** | **4.1 (0.62)** | **4.3 (0.53)** | **NA** |
| P1 | Maintain professional values (well-kempt, up-to-date knowledge and skills) | 776 | 1 (0.1) | 14 (1.8) | 92 (11.9) | 378 (48.7) | 291 (37.5) | 4.2 (0.73) | ***4.4 (0.66)*** | ***4.5 (0.57)*** | ***3.7 (0.70)*** |
| P2 | Portrait a good role model to others (punctual, polite, honest, trust-worthy etc.) | 775 | 5 (0.6) | 19 (2.5) | 154 (19.9) | 359 (46.3) | 238 (30.7) | 4.0 (0.81) | ***4.2 (0.91)*** | ***4.3 (0.69)*** | ***3.7 (0.69)*** |
| P3 | Communicate effectively with you and the other healthcare workers | 776 | 3 (0.4) | 40 (5.2) | 131 (16.9) | 370 (47.7) | 232 (29.9) | 4.0 (0.84) | ***4.1 (0.94)*** | ***4.2 (0.72)*** | ***3.7 (0.73)*** |
| P4 | Earn wide respect from his/her clinic staff | 765 | 2 (0.3) | 20 (2.6) | 157 (20.5) | 360 (47.1) | 226 (29.5) | 4.0 (0.79) | ***4.2 (0.85)*** | ***4.2 (0.72)*** | ***3.6 (0.64)*** |
| P5 | Improve the healthcare delivery system (appointment, reminder system, audit project etc.) At the clinic | 526 | 3 (0.6) | 13 (2.5) | 109 (20.7) | 273 (51.9) | 128 (24.3) | 4.0 (0.78) | 4.0 (0.83) | 4.0 (0.71) | NA |
| P6 | Encourage team work | 526 | 1 (0.2) | 13 (2.5) | 81 (15.4) | 255 (48.5) | 176 (33.5) | 4.1 (0.77) | 4.1 (0.83) | 4.1 (0.69) | NA |
| P7 | Contribute in the multi-disciplinary team effort in the clinics and community | 775 | 7 (0.9) | 39 (5.0) | 188 (24.3) | 381 (49.2) | 160 (20.6) | 3.8 (0.84) | ***4.0 (0.86)*** | ***4.0 (0.76)*** | ***3.5 (0.80)*** |
| P8 | Use available resources appropriately | 528 | 1 (0.2) | 8 (1.5) | 95 (18.0) | 299 (56.6) | 125 (23.7) | 4.0 (0.70) | 4.0 (0.74) | 4.0 (0.66) | NA |
|  | **Professionalism and Team-work** |  |  |  |  |  |  | **4.0 (0.68)** | **4.1 (0.71)** | **4.2 (0.56)** | **3.6 (0.68)** |
| R1 | Involve in research activity | 760 | 3 (0.4) | 45 (5.9) | 348 (45.8) | 270 (35.5) | 94 (12.4) | 3.5 (0.80) | ***3.7 (0.84)*** | ***3.7 (0.75)*** | ***3.2 (0.65)*** |
| R2 | Perform medical audits | 290 | 2 (0.7) | 14 (4.8) | 88 (30.3) | 128 (44.1) | 58 (20.0) | 3.8 (0.84) | ***3.8 (0.84)*** | ***3.2 (0.75)*** | NA |
| R3 | Provide feedback | 243 | 1 (0.4) | 3 (1.2) | 61 (25.1) | 124 (51.0) | 54 (22.2) | 3.9 (0.75) | 3.9 (0.75) | NA | NA |
| R4 | Involve in scientific writing and publication | 736 | 12 (1.6) | 55 (7.5) | 419 (56.9) | 199 (27.0) | 51 (6.9) | 3.3 (0.77) | ***3.4 (0.84)*** | ***3.5 (0.79)*** | ***3.1 (0.64)*** |
|  | **Ethical Practice** |  |  |  |  |  |  | **3.5 (0.71)** | **3.7 (0.68)** | **3.6 (0.69)** | **3.1 (0.62)** |

*Reverse score mean (SD)

HC= health clinics; HO= health offices; HP= hospitals

Bold and italic under Mean (SD) columns indicate significant difference at *p* value < 0.05
