# Supplementary material for: A nationwide postal survey on the perception of Malaysian public healthcare providers on family medicine specialists’ (PERMFAMS) clinical performance, professional attitudes and research visibility
Source: Springerplus. 2015 May 6;4:213. doi: 10.1186/s40064-015-1004-9 (PMC4431984; doi:10.1186/s40064-015-1004-9)
Supplement: Additional file 2: — Internal Consistency (Cronbach’s Alpha) for the Items according to the Domains and Public Healthcare Facilities. [file 40064_2015_1004_MOESM2_ESM.docx]

**Additional file 2: Internal Consistency (Cronbach’s Alpha) for the Items according to the Domains and Public Healthcare Facilities**

| **Domains and Items** | | | **Health Clinic** | **Health Office** | **Hospital** | **Overall*** |
| --- | --- | --- | --- | --- | --- | --- |
| **Clinical Competency** | | | **C1-C15** | **C1,C3,C4,C12,C13,C15** | **C1-C3,C5,C12,**  **C13,C14,C16,C17** | **C1,C2, C12,C13** |
| C1 | Improve the overall health of patients under his/her care. | | 0.89 | 0.77^‡^ | 0.85 | 0.66^§^ |
| C2 | Manage patient appropriately. | |  |  |  |  |
| C3 | Practise evidence-based care for his/her patients. | |  |  |  |  |
| C4 | Have a positive mind-set towards clinical practice guidelines. | |  |  |  |  |
| C5 | Empower patients to involve in their own care. | |  |  |  |  |
| C6 | Practice continuity of care (often see back/follow-up his/her patients). | |  |  |  |  |
| C7 | Allow walk-in consultation from patient. | |  |  |  |  |
| C8 | Allow ad-hoc consultation from MOs/allied health personals | |  |  |  |  |
| C9^†^ | Have long waiting time for new referral (more than one month) | |  |  |  |  |
| C10 | Does perform relevant procedures i.e. IUCD, Paps smear, ultrasound etc. | |  |  |  |  |
| C11 | Home visit to needy patient. | |  |  |  |  |
| C12 | Often receive compliment from patients. | |  |  |  |  |
| C13^†^ | Often receive complaints from patients. | |  |  |  |  |
| C14 | Make appropriate pre-referrals preparation of patient for what to expect at the hospital. | |  |  |  |  |
| C15 | Manage their time efficiently. | |  |  |  |  |
| C16 | Refer appropriate cases for further management (timely, work-up etc.). | |  |  |  |  |
| C17 | Write appropriate referral letter (adequate information, attachment etc.). | |  |  |  |  |
| **Ethical practice** | | | **E1-E4** | **E1-E4** | **E1,E2** | **E1,E2** |
| E1 | | Uphold patient welfare. | 0.89 | 0.88 | 0.86 | 0.85 |
| E2 | | Respect for patient’s autonomy/decision. |  |  |  |  |
| E3 | | Ensure patient’s confidentiality. |  |  |  |  |
| E4 | | Treat all patients equally irrespective of their social status. |  |  |  |  |
| **Safe practice** | | | **S1-S4** | **S1,S2** | **NA** | **S1,S2** |
| S1 | | Ensure patient safety in treatment. | 0.89 | 0.86 | NA | 0.87 |
| S2 | | Adopt safety practices (e.g. universal precaution) at the clinic. |  |  |  |  |
| S3 | | Provide clinical input to rectify events as in incident reports. |  |  |  |  |
| S4 | | Implementing measures to improve patient’s safety (checklists etc.). |  |  |  |  |
| **Professionalism and team-work** | | | **P1-P8** | **P1-P7** | **P1-P4,P7** | **P1-P4,P7** |
| P1 | | Maintain professional values (well-kempt, up-to-date knowledge and skills). | 0.95 | 0.92 | 0.90 | 0.92 |
| P2 | | Portrait a good role model to others (punctual, polite, honest, trust-worthy etc.). |  |  |  |  |
| P3 | | Communicate effectively with you and the other healthcare workers. |  |  |  |  |
| P4 | | Earn wide respect from his/her clinic staff. |  |  |  |  |
| P5 | | Improve the healthcare delivery system (appointment, reminder system, audit project etc.) At the clinic. |  |  |  |  |
| P6 | | Encourage team work. |  |  |  |  |
| P7 | | Contribute in the multi-disciplinary team effort in the clinics and community |  |  |  |  |
| P8 | | Use available resources appropriately. |  |  |  |  |
| **Research and publication** | | | **R1-R4** | **R1,R4** | **R1,R4** | **R1,R4** |
| R1 | | Involve in research activity. | 0.77 | 0.75 | 0.94 | 0.80 |
| R2 | | Perform medical audits |  |  |  |  |
| R3 | | Provide feedback |  |  |  |  |
| R4 | | Involve in scientific writing and publication. |  |  |  |  |

*Items that are common for all three public healthcare facilities

^†^Reverse scoring was done prior analyses

^‡^Cronbach’s alpha improved to 0.83 if C13 was deleted

^§^ Cronbach’s alpha improved to 0.76 if C13 was deleted
